# Resistance to Bleomycin in Cancer Cell Lines Is Characterized by Prolonged Doubling Time, Reduced DNA Damage and Evasion of G2/M Arrest and Apoptosis

**DOI:** 10.1371/journal.pone.0082363

**Published:** 2013-12-04

**Authors:** Qi Wang, Kangping Cui, Osvaldo Espin-Garcia, Dangxiao Cheng, Xiaoping Qiu, Zhuo Chen, Malcolm Moore, Robert G. Bristow, Wei Xu, Sandy Der, Geoffrey Liu

**Affiliations:** 1 Department of Applied Molecular Oncology, Ontario Cancer Institute/Princess Margaret Hospital, Toronto, Ontario, Canada; 2 Department of Pharmacology and Toxicology, University of Toronto, Toronto, Ontario, Canada; 3 Department of Statistics and Actuarial Science, University of Waterloo, Waterloo, Ontario, Canada; 4 Department of Biostatistics, Princess Margaret Hospital, Toronto, Ontario, Canada; 5 Institute of Virology, Medical School of Wuhan University, Wuhan, Hubei, China; 6 Department of Medicine and Pharmacology, University of Toronto, Toronto, Ontario, Canada; 7 Division of Medical Oncology and Hematology, Princess Margaret Hospital, Toronto, Ontario, Canada; 8 Departments of Medical Biophysics and Radiation Oncology, University of Toronto, Toronto, Ontario, Canada; 9 Dalla Lana School of Public Health, University of Toronto, Toronto, Ontario, Canada; Wayne State University School of Medicine, United States of America

## Abstract

**Background:**

To establish, characterize and elucidate potential mechanisms of acquired bleomycin (BLM) resistance using human cancer cell lines. Seven BLM-resistant cell lines were established by exposure to escalating BLM concentrations over a period of 16-24 months. IC_50_ values and cell doubling times were quantified using a real time cytotoxicity assay. COMET and γ-H2AX assays, cell cycle analysis, and apoptosis assessment further investigated the mechanisms of BLM resistance in these cell lines.

**Results:**

Compared with parental cell lines, real time cytotoxicity assays revealed 7 to 49 fold increases in IC_50_ and a mean doubling time increase of 147 % (range 64 %-352%) in BLM-resistant sub-clones (p<0.05 for both). Higher maintenance BLM concentrations were associated with higher IC_50_ and increased doubling times (p<0.05). Significantly reduced DNA damage (COMET and γ-H2AX assays), G2/M arrest, and apoptosis (p<0.05 for each set of comparison) following high-dose acute BLM exposure was observed in resistant sub-clones, compared with their BLM-sensitive parental counterparts. Three weeks of BLM-free culturing resulted in a partial return to BLM sensitivity in 3/7 BLM-resistant sub-clones (p<0.05).

**Conclusion:**

Bleomycin resistance may be associated with reduced DNA damage after bleomycin exposure, resulting in reduced G2/M arrest, and reduced apoptosis.

## Introduction

Bleomycin (BLM) is a glycopeptide antibiotic isolated from *Streptomyces verticillis* [1,2]. As a chemotherapeutic agent, it is used in the treatment of multiple tumors, including but not limited to testicular carcinomas, lymphomas, and head and neck cancers [3,4]. Although the full pathway of the drug’s mechanism of action has not been elucidated, BLM does bind to iron and oxygen to produce reactive oxygen species (ROS) [5] that induces single- and double-strand DNA breaks, with the latter being primarily responsible for its anti-tumor effects [6,7]. It also causes lipid peroxidation and mitochondrial DNA damage [8]. Extended cell-cycle arrest/senescence, apoptosis and mitotic cell death are the most common cellular responses to BLM treatment [9]. 

BLM was found to induce G2/M cell cycle arrest in cancer cell lines [10,11]. This may be explained by a G2/M checkpoint response to DNA damage. The G2/M checkpoint is important for genomic stability, for it ensures that chromosomes are intact and ready for separation before cells enter mitosis [12]. Unlike the G1 checkpoint, G2/M checkpoint genes are often not mutated in cancer cells [13]. 

Resistance to BLM is a clinical concern, and typically occurs during relapse in germ cell tumors, where BLM is most commonly used clinically. Although the mechanism of BLM-resistance is unclear, several possibilities have been put forward, including: (a) altered BLM intake and efflux [14,15]; (b) elevated antioxidant level [5,11]; (c) enhanced repair ability for BLM-induced DNA damage [14,16,17]; and (d) increased metabolism (inactivation) of BLM [17–19].

The development of BLM resistance serves as an important mechanism for the evasion of chemotherapeutic eradication in cancer cells. However, the mechanisms responsible for acquired BLM resistance in human tumor cells have not been well investigated. In this study, we established BLM-resistance in seven human cancer cell lines, including lines of tumor types currently treated with BLM and others known to be either sensitive or resistant to BLM. Moreover, we characterized these cell lines with regard to their level of BLM-resistance, BLM-induced DNA damage, doubling time, cell cycle distribution, and degree of apoptosis (before and after BLM treatment) to increase our understanding of the potential mechanisms of resistance.

## Materials and Methods

### Cells and cell culture

Seven commercially-available human cancer cell lines with wide differences in innate sensitivity/resistance to BLM (HOP62, ACHN, NT2/D1, SF-295, NCCIT, NCI-H322M, and MBA-MB-231) were chosen from National Cancer Institute (NCI) or American Type Culture Collection (ATCC) [20]. Two (NT2/D1, NCCIT) were testicular cell lines (Table 1). 

**Table 1:** Description of Cell Lines.

Cell line	Abbreviation (Parental/Resistant)	Derived from which cancer type	Parental IC_50_(µg /ml) *
ACHN	ACHN_0_	Renal cell carcinoma	0.009
	ACHN_0.25_		
HOP-62	HOP_0_	Lung adenocarcinoma	0.11
	HOP_0.05_		
SF-295	SF_0_	CNS glioblastoma	0.14
	SF_0.4_		
NT2/D1	NT2_0_	Germ cell carcinoma	N/A
	NT2_0.1_		
NCCIT	NCCIT_0_	Germ cell carcinoma	N/A
	NCCIT_1.5_		
NCI-H322M	H322M_0_	Lung adenocarcinoma	25.8
	H322M_2.5_		
MDA-MB-231	MB231_0_	Breast adenocarcinoma	27.9
	MB231_3.0_		

Note: Cell lines with subscript “0” indicate parental (control) lines (e.g.,HOP_0_). The resistant sub-clones have a subscript identifying its maintenance BLM concentration, in µg/ml (e.g., HOP_0.05_). *Data obtained from NCI-60 drug screening panel [20].

NT2/D1 was maintained in Dulbecco’s Modified Eagle’s Medium (DMEM). Other lines were cultured in RPMI 1640. The conditions were 10% fetal bovine serum (FBS), 1% penicillin/streptomycin at 37°C in 5% CO_2_. Cells were grown as monolayers in 75 cm^2^ cell culture flasks unless otherwise stated. All cell lines tested negative for mycoplasma contamination by Polymer Chain Reaction (PCR) methods [21]. Cell lines were authenticated using Short Tandem Repeats (STR) testing [22]. 

### Establishment of bleomycin-resistant sub-clones from parental (control) cell lines

To develop BLM-resistance, cells were continually exposed to stepwise increases in the concentration of BLM over a period of 16 to 24 months. Briefly, cells were seeded at a density of ~5 ×10^5^/ml in a T75 cell culture flask with 10ml complete growth medium. After 4-6 hours of incubation, relatively low concentrations of BLM (ranging from 0.01 to 0.1µg/ml depending on the innate BLM-sensitivity), dissolved in phosphate-buffered saline (PBS) without Ca^2+^ and Mg^2+^, were added into the medium. Cells were left in BLM for 2 to 4 weeks or until a stable cell re-population formed. Regular medium replenishment was performed throughout this period. The BLM concentration was then increased by 0.5 to 2 fold. This stepwise dose escalation continued for 16 to 24 months until the BLM concentration reached at least ten times the starting concentration. Thereafter, all BLM-resistant cell lines (“BLM-resistant sub-clones”) were maintained in their highest achieved BLM concentration (“maintenance dose”). At the same time, regular passage of the parental cell lines were performed in parallel with the BLM-resistance establishment process. 

### BLM sensitivity test and doubling time analysis

Prior to the BLM resistance/sensitivity assessment (cytotoxicity assay), a cell proliferation assay was carried out on the xCELLigence system (Roche, USA) to identify the optimal conditions under which the real-time cytotoxicity assay should be running. Specifically, the proliferation assay was performed: a) to identify the optimal seeding density for the cytotoxicity assay for each of the cell lines; and b) to determine the duration of the cytotoxicity assay. 

The proliferation assay was carried out by seeding various numbers of cells into a 96-well plate (E-plate 96, Roche, USA) in quadruplicate, followed by real time monitoring of cellular growth for up to 7 days. Twenty-four hours after the seeding, half of the wells on the plate were treated with BLM to determine the cellular response. The proliferation assay was repeated twice on each cell line. Optimal seeding densities for each line were selected on the basis of dramatic changes in proliferation at 72-96 hours after BLM treatment and small variations across replicate wells. 

For cytotoxicity assays, the cell count was first performed, and cells were then seeded into triplicate wells with 180µl of BLM-free cell culture medium on a 96-well plate. Twenty-four hours after initial plating, 20µl of cell culture medium containing serially diluted BLM ranging from 0 to 800 µg/ml were added into each well. The number of viable live cells was monitored every 15 minutes, for a total of at least 120 hours. IC_50_ (integrated software, xCELLigence system) and fold differences in IC_50_ between BLM-resistant and parental (control) cell lines (IC_50 BLM-resistant_ / IC_50 control_) were subsequently calculated. The fastest growth period observed for each of the cell lines in the proliferation assay was isolated for doubling time determination and its percentage change was calculated using xCELLigence system software. 

### Comet assay assessment of BLM-induced DNA damage

BLM is known to cause DNA damage in cells [6,7]. To determine initial (baseline) and DNA strand breaks after high dose BLM expose, alkaline Comet assays (single-cell gel electrophoresis) were performed [23] for each of the parental and resistant sub-clones. Olive Tail Moment (OTM) values of one hundred cells were scored at random per slide using fluorescence microscope with KOMET 5.0 software (Kinetic Imagine). 

### BLM-induced γ-H2AX foci formation

DNA double-strand breaks (DSBs) triggers the cellular formation of γ-H2AX foci (phosphorylated H2AX protein) [24]. To confirm the cellular DNA damage response to BLM through the Comet assay, quantitative analysis of γ-H2AX foci formation following high dose BLM exposure was performed on a subset of four parental/resistant pairs (HOP, ACHN, NCCIT, and H322M) [25] using Phospho-Histone H2AX pSer139 Monoclonal Antibody and Alexa Flour 488-conjugated anti-phospho-H2AX (BioLegend, San Diego, CA, USA). A minimum of 10000 events were counted on flow cytometer for each measurement; the intensity of γ-H2AX, which directly correlates with cytometry counts, was analyzed using Cell Quest software (BD, USA).

### Cell cycle distribution analysis

Cell cycle distributions of each of pair of seven parental and resistant sub-clones were tested pre- and post- 24 hours of high dose BLM exposure at ten times the resistant sub-clones’ maintenance concentration. Then, cells in the mono-dispersed suspension were fixed with ethanol, followed by propidium iodide (PI) staining (PI, Sigma, USA) and analyzed using the FACScalibur flow cytometer (BD, USA). Percentages of cells resting in G1, S and G2/M phase were determined (CellQuest software, BD, USA and ModFit LT software, Verity Software House). Cell cycle distribution was measured in each parental/BLM-resistant pair at baseline and at different time points up to 24 hours of BLM treatment. Correlations between cell cycle distribution, IC_50_ values, and cell line doubling times were analyzed. 

### Annexin V/PI assay for BLM-induced apoptosis

To determine cell apoptosis pre- and post- BLM treatment, a representative subset of four parental/resistant pairs (HOP, ACHN, NCCIT, and H322M) was treated with 24 hours of high- dose BLM. The cells were then stained with Annexin V–FITC and PI, and evaluated for apoptosis by flow cytometry according to the manufacturer's protocol (BD PharMingen, San Diego, CA, USA). Both early (Annexin V-positive, PI-negative) and late apoptotic cells (Annexin V-positive, PI-positive) were counted as apoptotic cells.

### Statistical Analysis

To assess treatment significance or difference, paired T-tests or unpaired T-tests (depending on the experimental specifications) were performed with a two-sided significance level of 0.05. Normality assumptions were assessed via Shapiro-Wilks tests. When the normality assumption could not be held, paired or unpaired Wilcoxon rank-sum tests were performed instead. For Comet assay assessment between parental and resistant sub-clones after high-dose BLM treatment, p-values were calculated using a t statistic for non-equal variances, testing the null hypothesis of equality on log ratios. Logistic regression was performed to assess baseline G2/M distribution differences between parental and resistant sub-clones. To investigate correlation between various measures (IC_50 control_, IC_50_ change following high-dose BLM treatment, doubling time, and cell cycle distribution), linear regression analyses were performed. All analyses were conducted using SAS version 9.2 or SPSS version 13.0. 

## Results

### BLM-resistant cell lines maintained on BLM stably displayed higher IC_50_ values and prolonged doubling times

**Figure 1:**
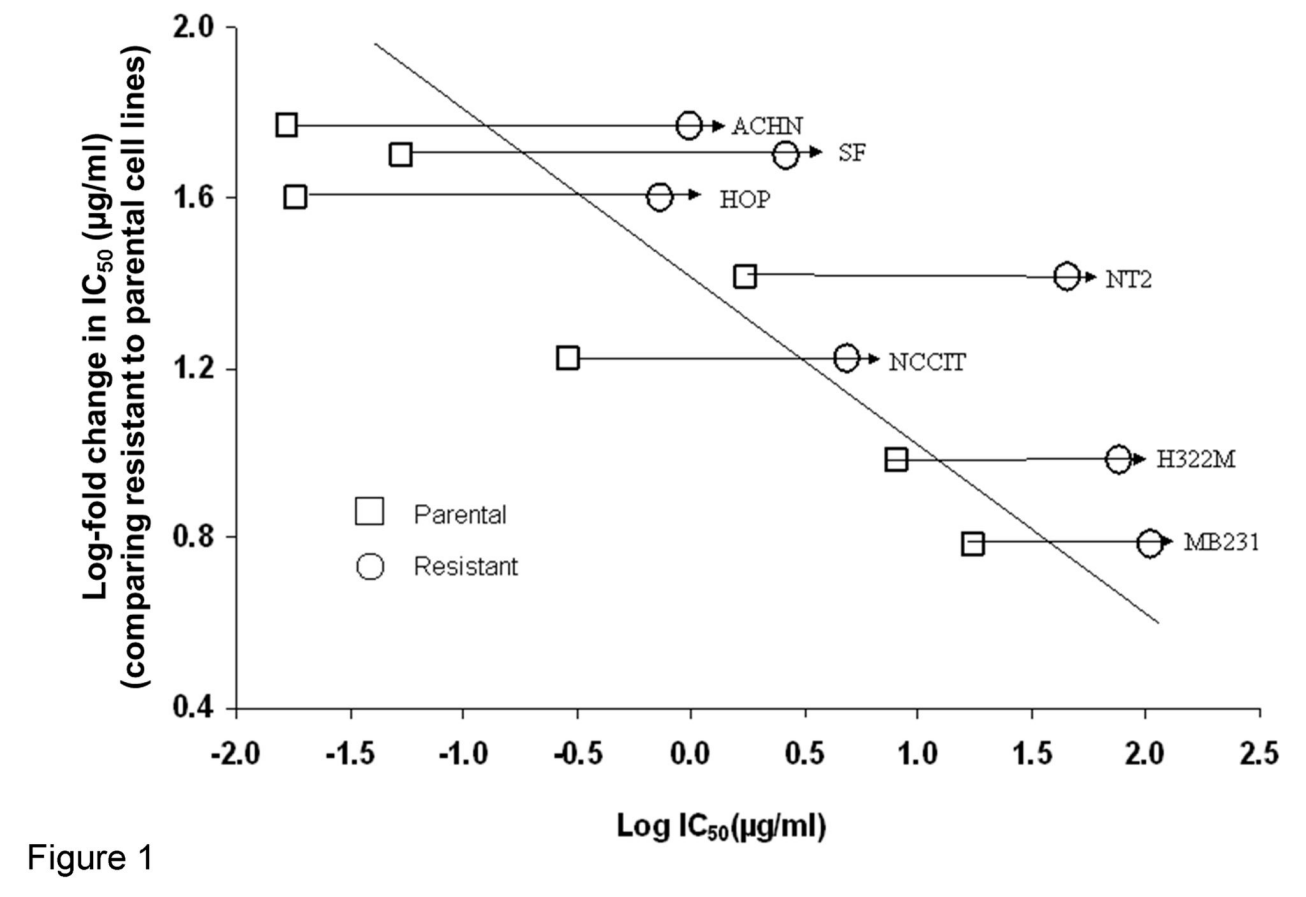
Correlation between IC_50_ fold increase and IC_50_ values of control cell lines. Linear regression models determined that higher values of IC_50_ were associated with lower values of fold change (logarithm scale slope of: -0.11 (standard error: 0.02), P < 0.0001, R^2^= 0.58). Each IC_50_ value is the mean of experiments performed in triplicate.

All seven BLM-resistant sub-clones demonstrated greater IC_50_ than their parental counterparts (Figure 1). Cytotoxicity assays showed between 7-49 fold increases of IC_50_ in BLM-resistant sub-clones. A positive correlation was observed between the maintenance BLM concentration and IC_50_ values (p<0.001, R^2^=0.58). After prolonged BLM exposure, cell lines with greater parental sensitivity to BLM (mean IC_50_, 0.1µg/ml) exhibited a greater increase in resistance (mean of 48 fold) compared to parental lines that were less sensitive (mean IC_50_, 9µg/ml, 15 fold; p<0.05 comparing parental sensitive to less sensitive lines). 

**Figure 2:**
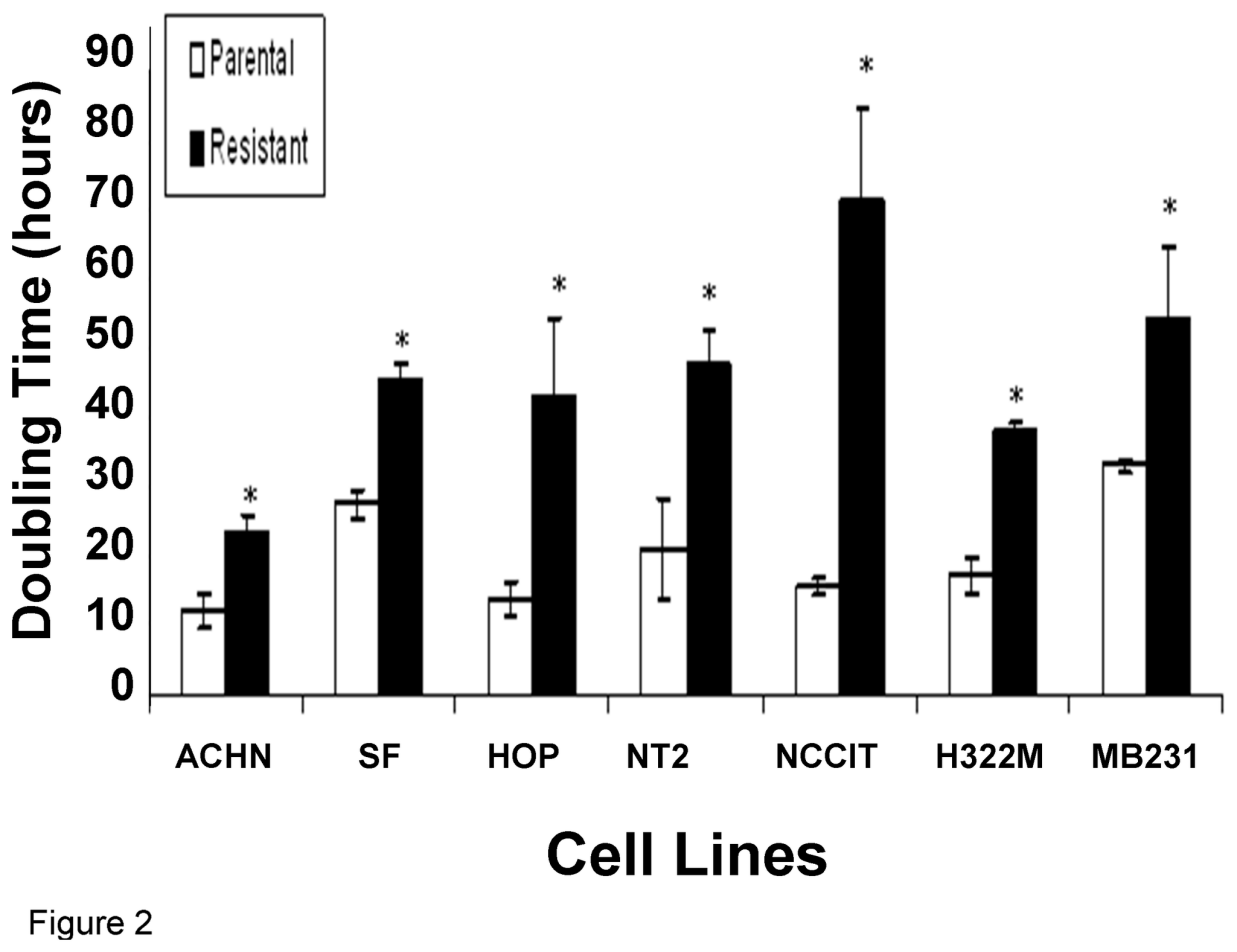
Average doubling time of parental (control) and BLM-resistant sub-clones. Mean doubling time ± standard error of the mean (SEM, n=3) was reported. The mean doubling time (measured in hours) of the parental lines was shorter than that of BLM-resistant sub-clones in all seven cell lines. * P<0.05 compared to parental.

It was observed that BLM-resistant sub-clones grew slower than their parental cell lines. Two cell lines, when maintained in higher concentrations of BLM, such as MB231_3.0_ and H322M_2.5_ (subscripts denote maintenance BLM concentration), also exhibited enlarged and flattened cell morphology resembling that of cell senescence compared to their parental lines, but only after many generations. In contrast, acute exposure to high doses of BLM did not result in morphological changes. The slower cellular growth was confirmed by cell doubling time calculated with the xCELLigence system. All BLM-resistant sub-clones displayed statistically significant doubling time prolongation with a mean doubling time increase of 147% (range: 64%-352%) when compared with their parental cell lines (Figure 2, p<0.05). There was no correlation between cell doubling time and IC_50_ values, and none between the percentage increase in doubling time and fold increase in IC_50_. 

**Figure 3:**
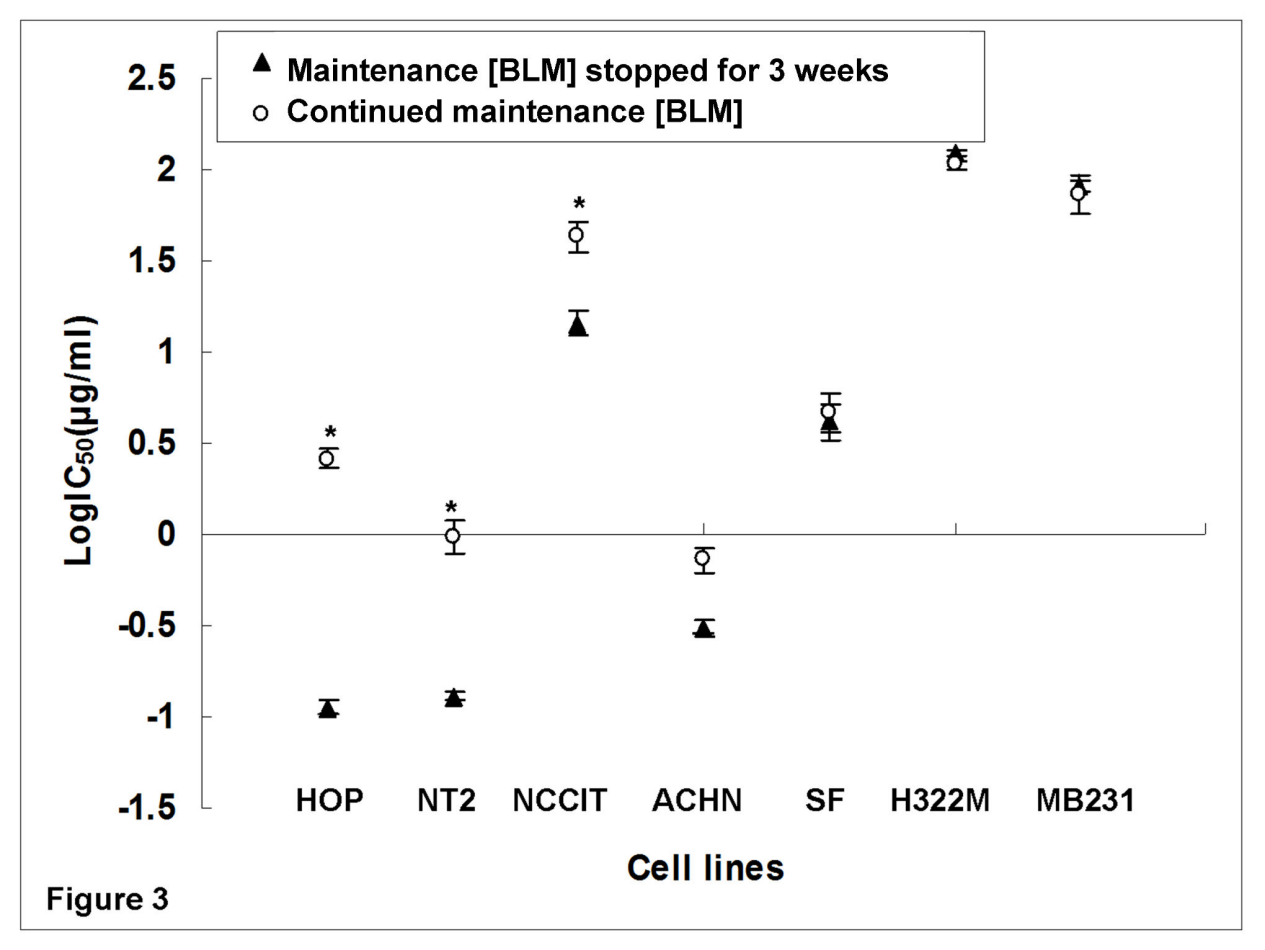
Effects of 3-week discontinuation of maintenance BLM treatment on IC_50_ (µg/ml). Experiments were performed in triplicate. Log IC_50_ comparisons were performed. Three (HOP_0.05_, NT2_0.1_, and NCCIT_1.5_) of the seven cell lines had significant reductions in IC_50_ values following three weeks of BLM-free maintenance. * P<0.05 for comparisons between BLM resistant sub-clones and their corresponding counterparts with three weeks of treatment break.

**Figure 4:**
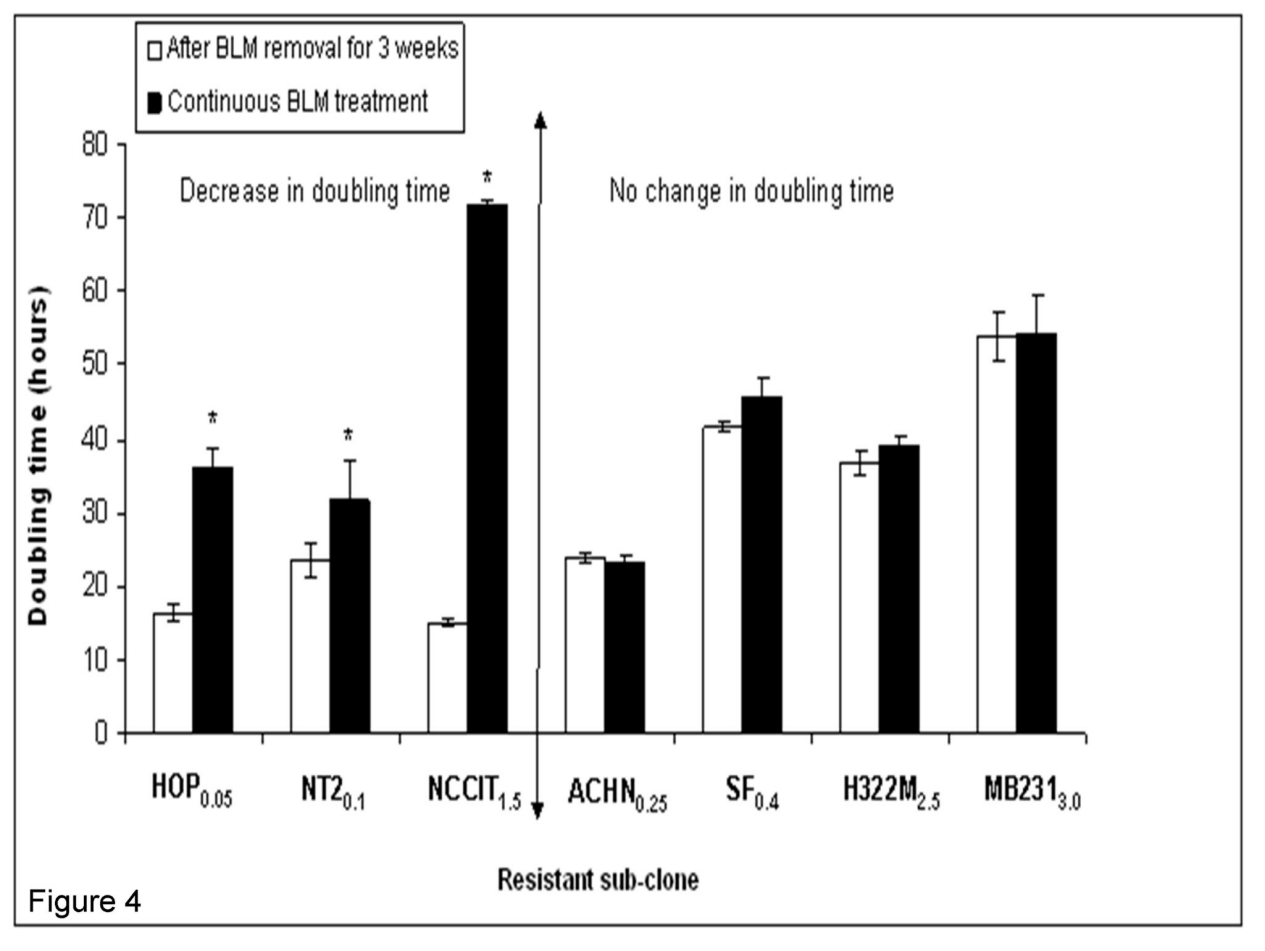
Effects of 3-week discontinuation of maintenance BLM treatment on doubling time. Experiment was performed in triplicate. Three (HOP_0.05_, NT2_0.1_, NCCIT_1.5_) of the seven cell BLM-resistant lines exhibited significant decrease in doubling time following three weeks of BLM-free treatment. * P<0.05 compared to after removal BLM for 3 weeks cell lines.

To test the stability of BLM resistance in BLM-resistant sub-clones, comparisons in IC_50_ values and doubling times were made between normally maintained BLM-resistant sub-clones and the same resistant sub-clones which were subsequently cultured in BLM-free medium for three weeks. After three weeks of BLM-free culturing, three of the originally resistant sub-clones (including both testicular cell lines NT2_0.1_, NCCIT_1.5_ and the lung cancer cell line HOP_0.05_) exhibited a significant IC_50_ reduction (Figure 3) and doubling time reduction (Figure 4), when compared to regularly maintained BLM-resistant sub-clones. There were no statistically significant changes in IC_50_ and doubling time in the remaining four lines.

### BLM resistance may be dose-dependent

Given that a general correlation exists between IC_50_ values and the maintenance BLM concentrations across 7 cell lines (Figure 1), the possibility of dose-dependent BLM resistance was tested. For the single cell line ACHN, IC_50_ values were obtained from ACHN_0_ (parental line), and its two resistant sub-clones, ACHN_0.1_ and ACHN_0.25_. A positive correlation was found between the maintenance BLM concentrations (0, 0.1 and 0.25µg/ml) and their IC_50_ values (0.01, 0.29, and 0.74µg/ml respectively, p<0.05). Moreover, a positive correlation was also observed between BLM maintenance concentrations and doubling times (0µg/ml-12hrs, 0.1µg/ml-16.5hrs, 0.25µg/ml-23.5hrs, p<0.05). This finding was not tested or confirmed in any of the other cell lines.

### BLM-resistant sub-clones had less BLM-induced DNA damage in Comet assays

**Figure 5:**
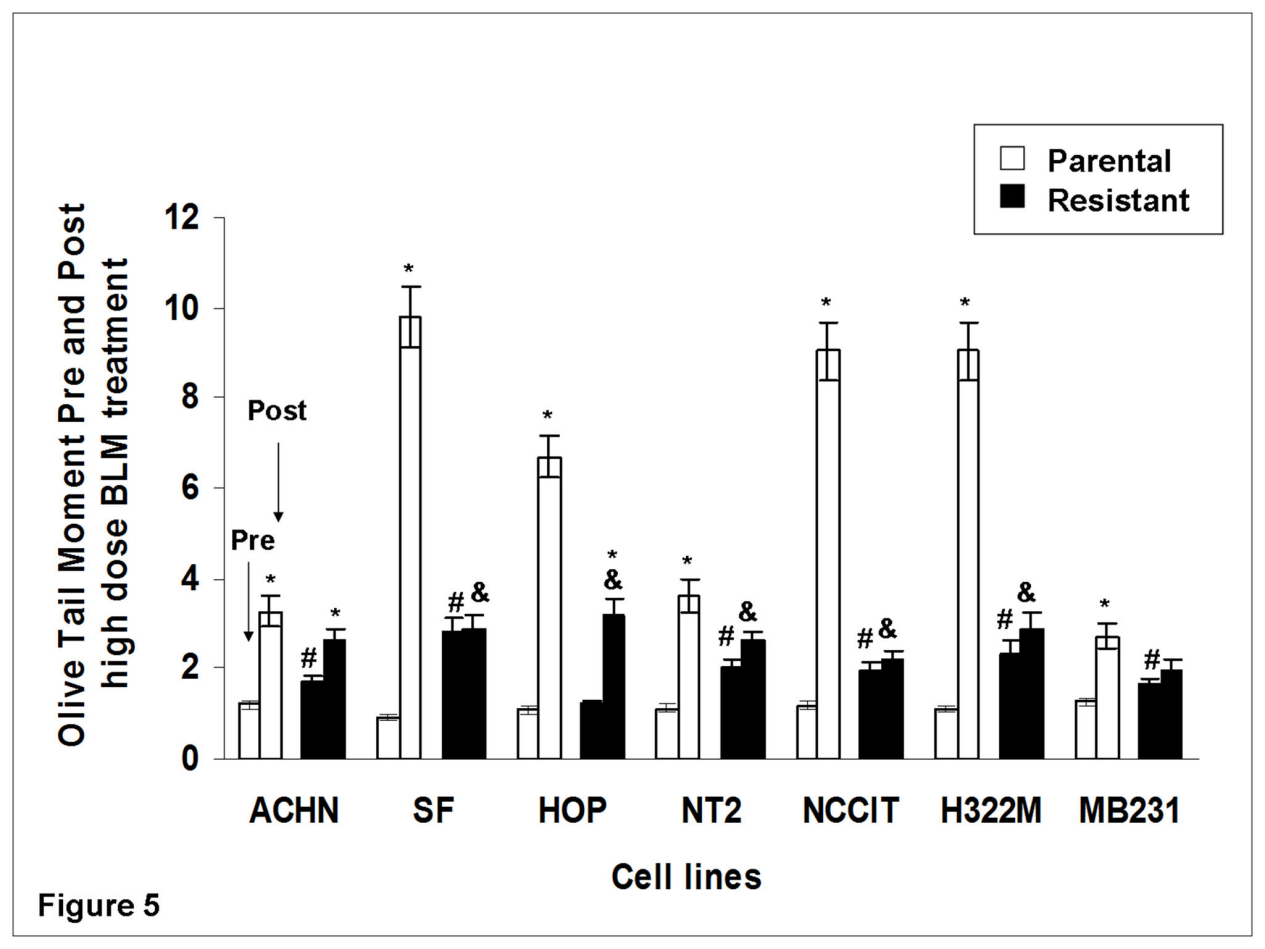
DNA damage in Olive Tail Moment (OTM) pre- and post- high dose BLM treatment assessed by comet assay. Experiments were run in triplicates. Cells were subject to high dose BLM exposure (corresponding to ten times their respective maintenance concentrations) for 24 hours. OTM was used for DNA fragmentation (damage) quantification, and was calculated as: OTM = (Tail.mean - Head.mean) × (Tail %DNA)/100. Comet assay revealed greater increase in DNA fragmentation (expressed in OTM levels) after BLM treatment in all parental lines.* P<0.05 for comparison between cell lines prior and after high dose BLM treatment. All parental lines exhibited significant increase in DNA damage. # P<0.05 for comparison between parental and resistant cell lines at baseline (pre-treatment). All BLM-resistant lines except for HOP_0.05_ exhibited increased DNA damage at baseline compared to their parental lines. & P<0.05 for comparison between resistant cell lines and parental cell line post BLM treatment. Less DNA damage (compared to their parental lines) post- BLM treatment was found in five of seven BLM-resistant cell lines (SF_0.4_, HOP_0.1_, NT2_0.1_, NCCIT_1.5_, and H322M_2.5_).

Quantification of DNA damage in all seven parental/resistant pairs using Comet assay (measured in OTM) showed that prior to BLM treatment, six of the seven resistant cell lines had higher basal DNA damage compared with control (the exception was HOP_0.05_, p<0.05). This generally correlated with the prolonged basal cell doubling time observed in these resistant sub-clones. Following high dose BLM treatment, five of seven resistant sub-clones (SF_0.4_, HOP_0.1_, NT2_0.1_, NCCIT_1.5_, and H322M_2.5_) had lower DNA damage than their parental lines. No increase in DNA damage after BLM exposure was observed in five of seven resistant lines (SF_0.4_, NT2_0.1_, NCCIT_1.5_, H322M_2.5_, and MB231_3.0_). In contrast, all parental cell lines had greater DNA damage post- BLM than pre- BLM (p<0.05 for each comparison; Figure 5). Further, all seven parental lines displayed significantly greater DNA damage increase post- BLM treatment when compared to their resistant counterparts (p<0.05). 

### BLM-resistant sub-clones had reduced γ-H2AX levels compared to their parental lines following high dose BLM treatment

**Figure 6:**
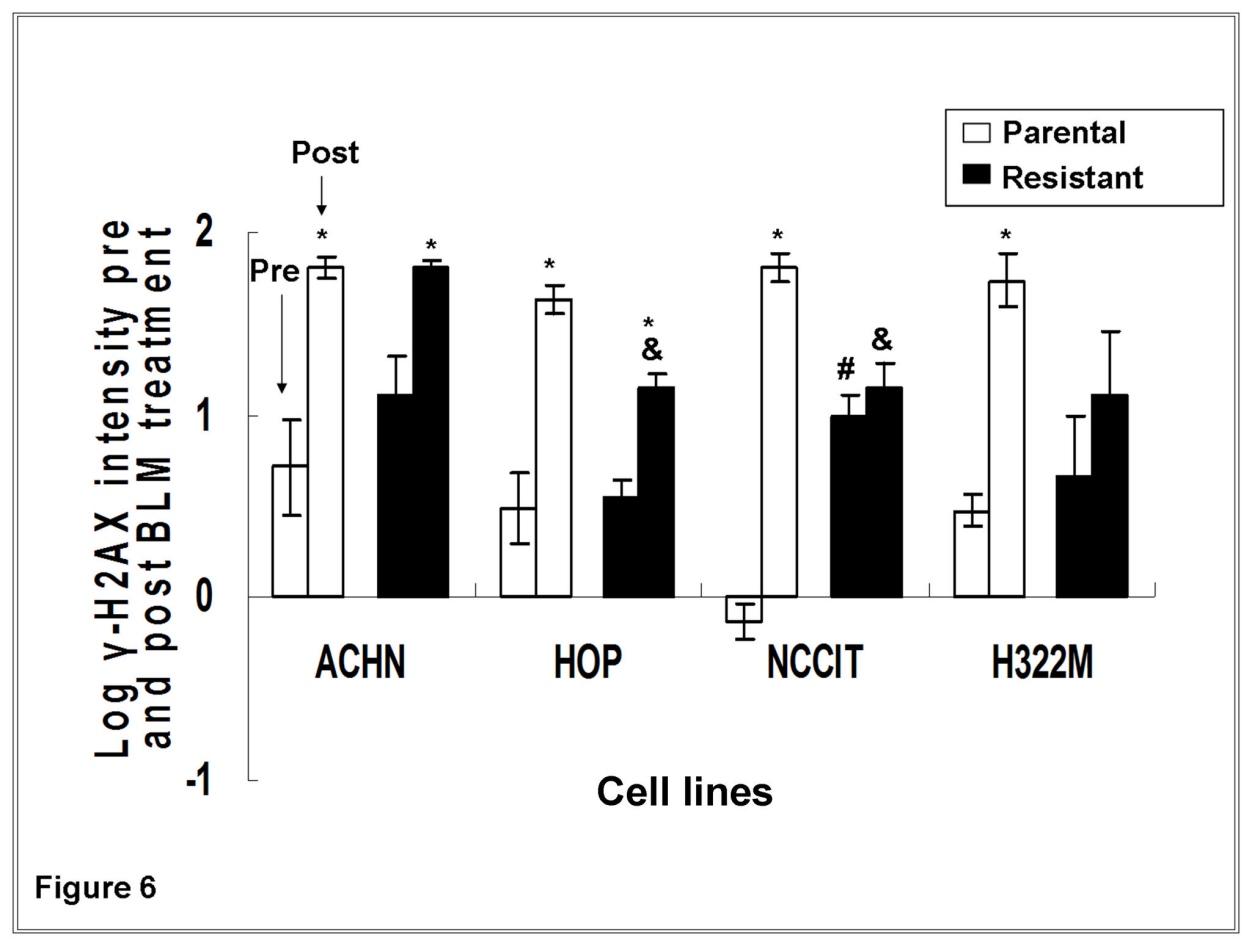
γ-H2AX formation pre- and post- high dose BLM treatment assessed by flow cytometry. Experiments were run in triplicate. Cells were subject to high dose BLM exposure (corresponding to ten times their respective maintenance concentrations) for 24 hours. Flow cytometric detection of BLM-induced γ-H2AX foci formation were then obtained in a subset of four cell lines (ACHN, HOP, NCCIT and H322M). * P<0.05 for comparison between cell lines prior and after high dose BLM treatment. All parental lines exhibited significant increase in formation of γ-H2AX. # P<0.05 for comparison between parental and resistant cell lines at baseline (pre-treatment). One of four BLM-resistant cell lines (NCCIT_1.5_) had greater γ-H2AX formation than its parental counterpart at baseline. & P<0.05 for comparison between resistant and parental cell lines following BLM treatment. Two of four BLM-resistant cell lines (HOP_0.1_ and NCCIT1._5._) revealed significantly less γ-H2AX formation than their parental counterparts post BLM treatment, with a third line (H322M_2.5_) being borderline significant (p=0.054).

As a second measure of cellular response to DNA damage, γ-H2AX was also assessed in a subset of four cell lines (HOP, ACHN, NCCIT and H322M). Following 24 hours of high dose BLM treatment, γ-H2AX intensities increased in all parental cell lines. In the resistant sub-clones, increased γ-H2AX intensities were only observed in two of four lines (ACHN_0.25_ and HOP_0.05_,Figure 6). This is in agreement with the Comet assays. Three (HOP_0.05_, NCCIT_1.5_, and H322M_2.5_) of the four resistant sub-clones exhibited significantly less change in γ-H2AX intensity (γ-H2AX intensity post-treatment minus pre-treatment) compared with their parental sub-clones post- BLM treatment (p<0.05). This trend was borderline significant in the fourth line (H322M_2.5_, p=0.054).

### BLM-resistant cell lines had a lower percentage of G2/M arrest following high dose BLM exposure

**Figure 7:**
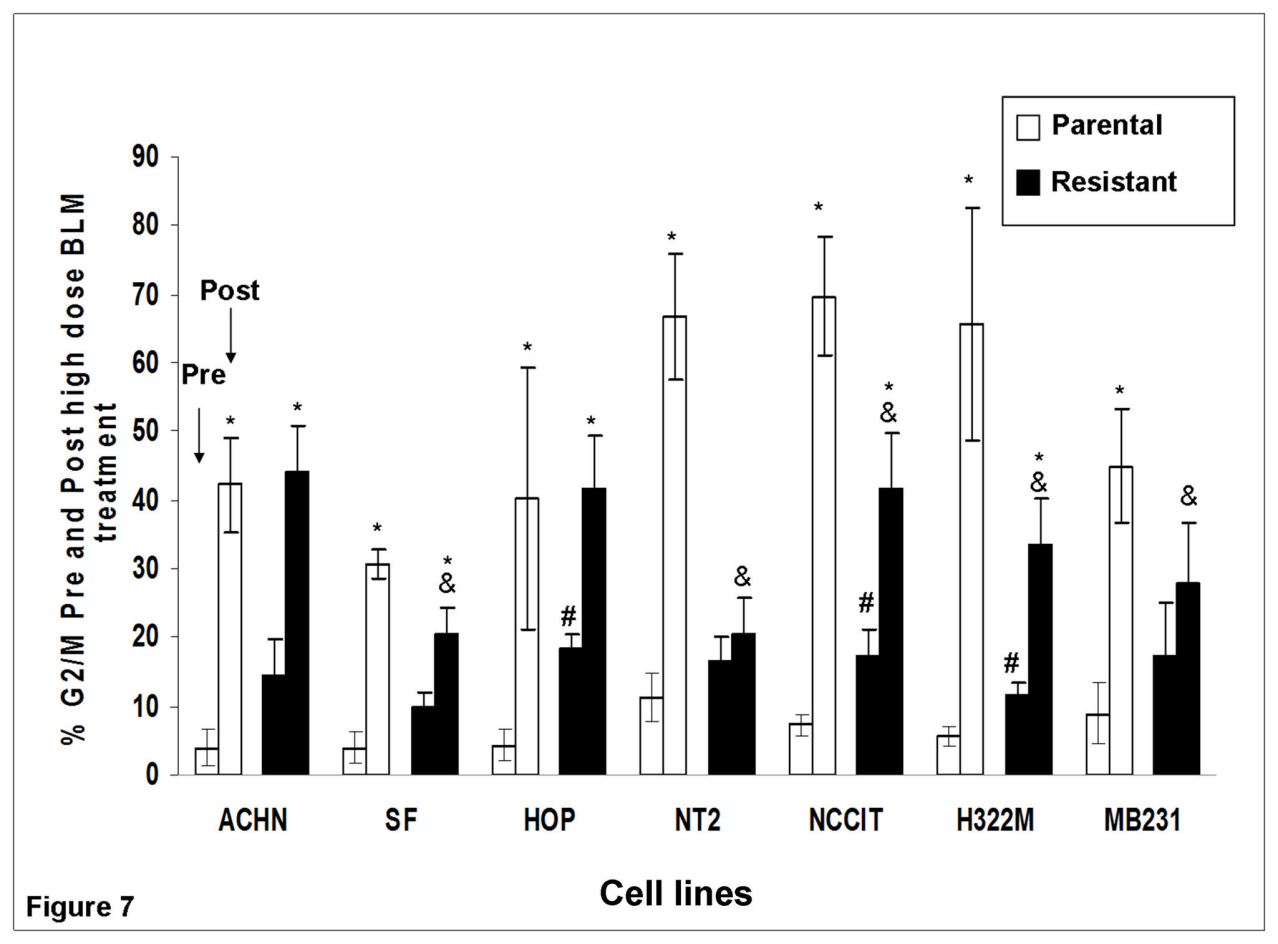
Summary of % G2/M phase distribution pre- and post- high dose BLM treatment in all 7 parental/resistant pairs. High dose BLM treatment corresponds to ten times the corresponding maintenance concentration for each cell line. Mean ± SEM (n=3) values are reported. * P<0.05 for the comparison between cell lines prior to and after high dose BLM treatment. All parental lines exhibited significant increases in G2/M cell cycle distribution. # P<0.05 for comparison between parental and resistant cell lines at baseline (pre-treatment). Three of seven BLM-resistant cell lines (HOP_0.05_, NCCIT_1.5_, and H322M_2.5_) exhibited increased % G2/M distribution at baseline compared to their parental cell lines. & P<0.05 for comparison of % G2/M distribution between parental and resistant cell lines after BLM treatment. Less % G2/M distribution than parental lines was found in five out of seven BLM-resistant cell lines (SF_0.4_, NT2_0.1_, NCCIT_1.5_, H322M_2.5_, and MB231_3.0_) after BLM treatment.

Since cell cycle arrest at G2/M phase was a characteristic general cellular response to BLM exposure, the ability of BLM-resistant sub-clones to suppress BLM-induced G2/M arrest was evaluated. As shown in Figure 7, three of seven BLM-resistant sub-clones (HOP_0.05_, NCCIT_1.5_, and H322M_2.5_) exhibited greater G2/M phase distribution at baseline, compared with their parental lines (p<0.05). Similarly, for the other four cell lines, the resistant sub-clones also exhibited greater G2/M phase distribution at baseline, though non-significantly. After 24 hours of high dose BLM exposure, five (SF_0.4_, NT2_0.1_, NCCIT_1.5_, H322M_2.5_, and MB231_3.0_) of seven BLM-resistant sub-clones exhibited a lower G2/M distribution than their corresponding parental lines (p<0.05). Comparing the % G2/M distribution before and after 24 hours of high dose BLM treatment, all parental cell lines exhibited increases in G2/M distribution following the treatment (p<0.05).The same trend was seen in all resistant sub-clones, although two (NT2_0.1_ and MB231_3.0_) were non-significant. The extent of % G2/M distribution increase (calculated as % G2/M_post-treatment_ minus % G2/M_pre-treatment_) was smaller for all resistant sub-clones than their corresponding parental lines (p<0.05). 

### G2/M arrest becomes prominent after 8 hours of high dose BLM treatment

**Figure 8:**
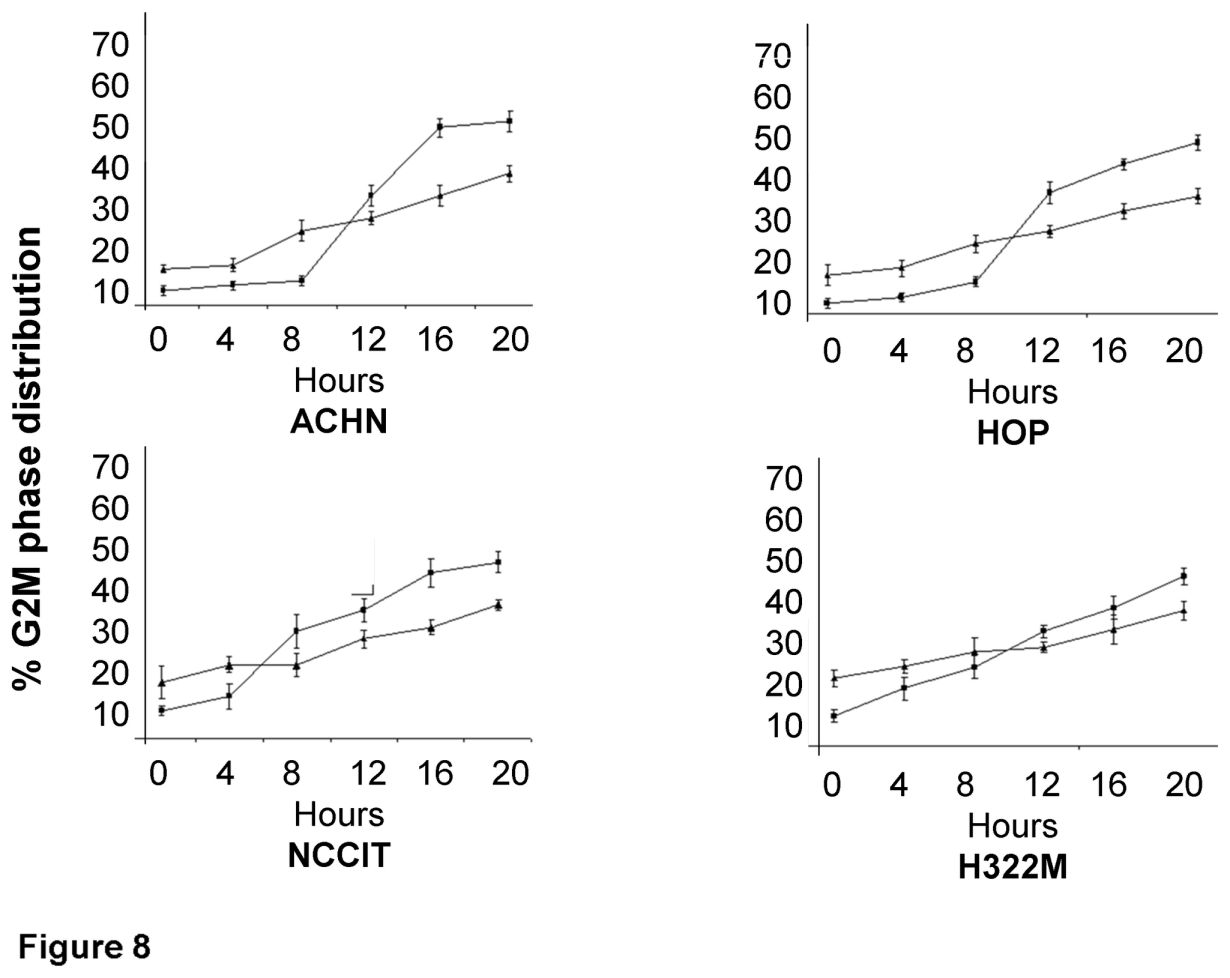
Time course measurement of % G2/M distribution in four parental/resistant cell line pairs at 0 (baseline) 4, 8, 12, 20, and 24 hours following high dose BLM treatment. Experiments were run in triplicate. G2/M distribution was found to be higher in parental lines (compared to resistant sub-clones) 8 hours after BLM treatment.

To evaluate the timing of G2/M arrest after high dose BLM exposure, four cell lines (the innately BLM sensitive HOP and ACHN lines, the NCCIT testicular line and the innately BLM resistant H322M, the same lines evaluated for the γ-H2AX experiment) underwent a time course analysis. Although there was increasing G2/M arrest in both parental and BLM-resistant sub-clones (Figure 8), this arrest was most prominently seen between 8 and 12 hours after BLM treatment in parental cells.

### Reduced apoptosis was found in BLM-resistant sub-clones

**Figure 9:**
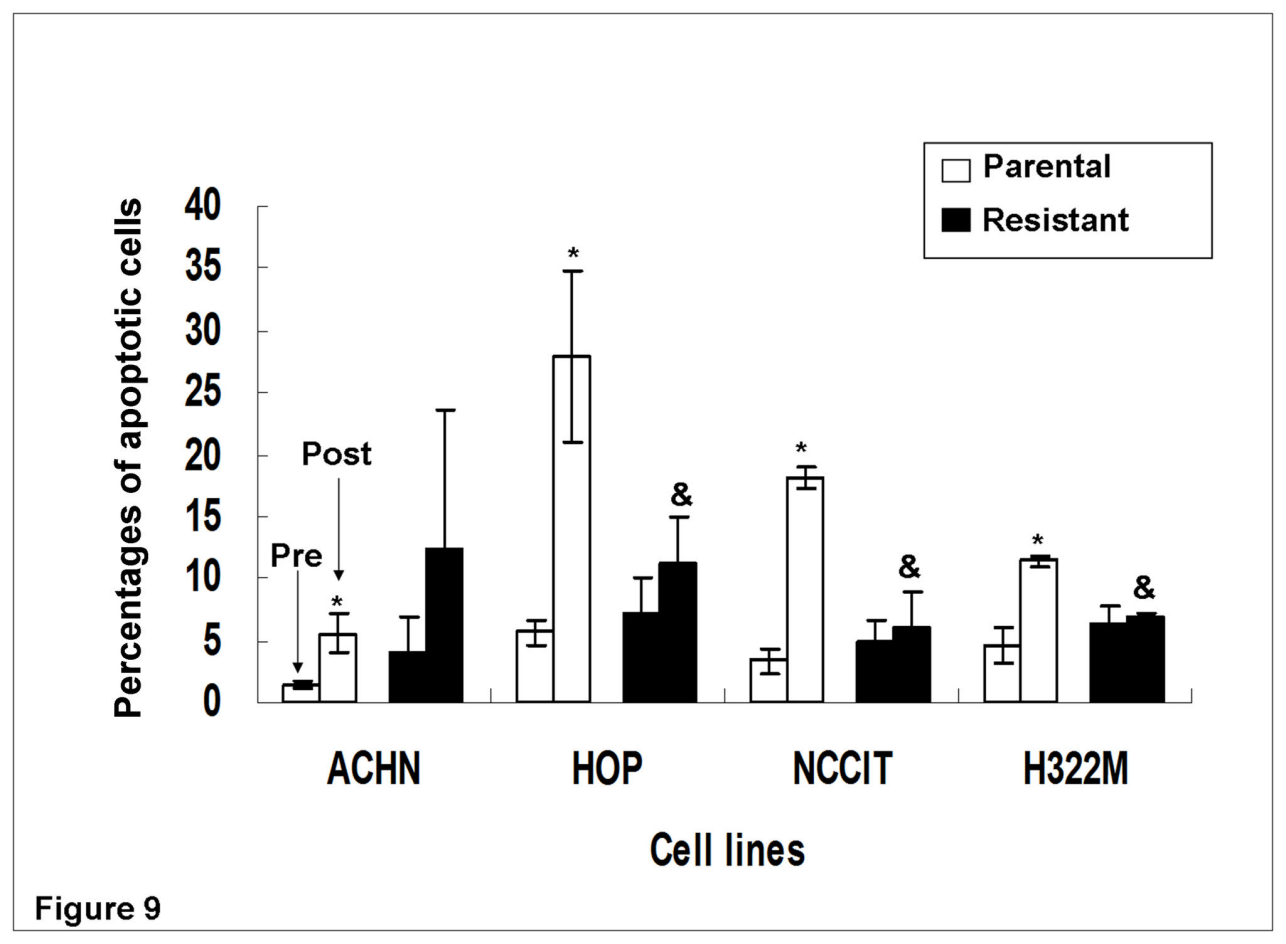
Percent cell apoptosis pre- and post- high dose BLM exposure in four parental/resistant cell line pairs. * P<0.05 for comparison between cell lines prior to and after high dose BLM treatment. All parental lines but no resistant lines exhibited significant increases in apoptosis post- BLM treatment. & P<0.05 for comparison between resistant and parental cell line following BLM treatment. Less cell apoptosis was found in three (HOP_0.05_, NCCIT_1.5_, and H322M_2.5_) of four BLM-resistant lines, when compared to their parental lines.

Using the same four paired sub-clones, we examined whether cells underwent apoptosis after high dose BLM treatment. After 24 hours of BLM treatment, the percentage of apoptotic cells increased in all four parental lines tested (Figure 9). In contrast, no resistant sub-clones exhibited statistically significant increases in apoptosis following BLM treatment. This was in agreement with the Comet assay (DNA damage) analysis. Moreover, three of four resistant sub-clones (HOP_0.05_, NCCIT_1.5_, and H322M_2.5_) exhibited significantly less increase in apoptosis (% apoptosis after minus % apoptosis before BLM treatment) compared with their parental lines following BLM treatment (p<0.05). This generally correlated with the reduced DNA damage and G2/M cell cycle arrest in BLM-resistant sub-clones (compared with parental lines, post-treatment) as observed previously. 

## Discussion

In this study, we successfully established seven BLM-resistant human cancer cell lines from commercially available cancer cell lines of various organ origins (lung, testicle, breast, kidney, as well as the central nervous system). After sudden acute, short-term exposure to BLM, these BLM-resistant sub-clones exhibited less DNA damage, had longer doubling times, had a lower proportion of cells in G2/M arrest, and had reduced apoptosis, when compared to their more BLM-sensitive parental cell lines. BLM-resistant cell lines were developed by progressively increasing the incubating BLM concentration over an extended period of 16-24 months. Previous studies may have utilized similar methods in cultivating BLM resistant sub-clones. However, few of them reached the high level of BLM-resistance observed in this study (which was 7-49 fold increase in IC_50_ between resistant and parental sub-clones, when compared to a 3-20 fold increase in another study [15]). Moreover, through analysis of BLM-induced DNA damage, cell cycle distribution, and percentages of apoptosis, several putative mechanisms of BLM resistance/sensitivity were evaluated. 

BLM is known to cause extended G2/M arrest and/or cell apoptosis [9] in BLM sensitive cells. This can be mediated by ATM/ATR [26,27], the upstream proteins of a DNA repair and signaling pathway that triggers G2/M arrest or cell apoptosis via a range of downstream gene products such as chk1/2, cdc 25 [28], p53, and p21^WAF1/CIP1^ , where the latter two are critical for sustaining G2/M cycle arrest [29]. Moreover, histone H2AX was found to be necessary for the activation of the G2/M check point [30]. 

Comet and γ-H2AX results revealed less BLM-induced DNA damage in the resistant lines, suggesting that the resistant sub-clones may have an enhanced ability to prevent and/or reduce DNA damage caused by BLM. This may be due to reduced cellular uptake of BLM, and/or enhanced BLM elimination/detoxification, mediated by cell surface receptors or transporters [31], antioxidant molecules/enzymes that reduce BLM-generated ROS [11,32]; or enzymes that inactivate BLM [33,34]. Future studies need to further study these mechanisms.

In this study, BLM resistance also resulted in less G2/M arrest and cell apoptosis, consistent with results from a previous study on a single BLM-resistant line [11]. The slight increase in G2/M arrest at baseline (prior to high dose BLM treatment) for these BLM resistant sub-clones could be explained by chronic exposure to BLM. The fact that BLM-resistant cells were able to proliferate at a BLM concentration that was not viable for their parental lines suggests that evasion of cell cycle blockage could be another mechanism of resistance. Taken together, our results suggest that BLM resistant cells may have acquired enhanced ability to prevent/reduce DNA damage caused by BLM. This in turn, may have led to the reduced G2/M arrest and apoptosis. 

A stepwise dose escalating method for BLM resistance development may result in a dose-response relationship among BLM maintenance concentrations, IC_50_ values and doubling times observed in ACHN_0_, AHCN_0.1_ and AHCN_0.25_ cells. However, until these findings are confirmed in other sets of sub-clones, this will remain an interesting finding in need of validation. 

After removing maintenance BLM concentrations from all seven resistant sub-clones, there was partial reversal of IC_50_ values and doubling time in three of seven cell lines. The absence of reversibility in the other four cell lines could be the result of individual cell line differences, or perhaps these cell lines needed longer breaks from BLM exposure to develop BLM sensitivity again. To our knowledge, the literature on reversing BLM-resistance in cancer cell lines has been extremely limited. This observation speaks to the potential importance of continued BLM exposure when developing resistant sub-clones. Moreover, the result suggests that reversible mechanisms such as epigenetic rather than (permanent) genetic changes may be playing a role in maintaining some of the BLM-resistance. 

This study has limitations. Firstly, an *in vitro* model may not explain resistance in patients, but remains a critical first step in studying BLM resistance mechanism. Secondly, the amount of BLM treatment for each cell line corresponded to ten times the maintenance concentration for each BLM resistant sub-clone. There is the possibility that the treatment is not “acute” or high enough to elicit significant cellular response following the treatment. However, significant responses were seen in parental cells. Thirdly, individual cell line differences were not well-accounted, given the range of cell line origins. Since each cell line may have unique mechanisms that contribute to BLM resistance, this may explain some of the variations observed across experiments in this study. Fourthly, the cells used were not monoclonal. This may result in cells behaving differently upon BLM treatment. Yet, given that it was technically difficult to repopulate cells from a single cell that survived the dose escalation, we adopted this conventional approach of resistance development. 

In summary, this study described some of the relationships between BLM resistance, BLM-induced DNA damage, cell growth rate, cell cycle distribution, and apoptosis. The reduced DNA damage, reduced G2/M arrest, and reduced apoptosis observed in BLM-resistant sub-clones following high dose BLM exposure suggest that acquired BLM resistance involves effective DNA damage reduction and G2/M cell cycle evasion. The seemingly reversible resistance observed in at least some of the BLM resistant sub-clones suggests that some of the BLM- resistance in our cell lines models may have utilized non-permanent mechanisms such as epigenetic changes to cope with chronic BLM exposure. Our results provide the foundation for future research in biomarkers of BLM resistance, which may ultimately lead to an improved rationale for personalized chemotherapy selection.
